# Linear scleroderma as a rare cause of enophthalmos: a case report

**DOI:** 10.1186/1752-1947-1-179

**Published:** 2007-12-14

**Authors:** Bertie S Fernando, Paul S Cannon, Krishna Tumuluri, Anne E Cook

**Affiliations:** 1 Department of Oculoplastics, Manchester Royal Eye Hospital, Oxford Road, Manchester, M13 9WH, UK

## Abstract

**Introduction:**

Enophthalmos is an important physical sign which can be easily missed.

**Case presentation:**

A 64-year old female presented with painless and progressive shrinking of her right eye. Visual acuity was 6/6 in both eyes. The main clinical findings included exophthalmometry readings of 14 mm in the right eye and 22 mm in the left eye and a linear scar on her right forehead. This scar is a feature of linear scleroderma and called "en coup de sabre". She was referred to a dermatologist for further assessment.

**Conclusion:**

Enophthalmos is defined as the relative recession of the globe into the bony orbit and if measuring greater than 2 mm can give a noticeable cosmetic deformity. Scleroderma is a systemic or localised disease. Linear scleroderma has the following features-localised fibrosis of the skin, blood vessels, subcutaneous fat, muscle and sometimes bone. Histology shows an inflammatory and a sclerotic phase. Ophthalmic effects include enophthalmos, lash loss, lid induration or tightening and periorbital oedema.

## Introduction

Enophthalmos is a subtle, frequently missed but important physical sign that can and should be accurately diagnosed. Distinction between the various causes of enophthalmos can be difficult. The treatment and prognosis differ considerably between the various causes.

## Case presentation

**Figure 1:**
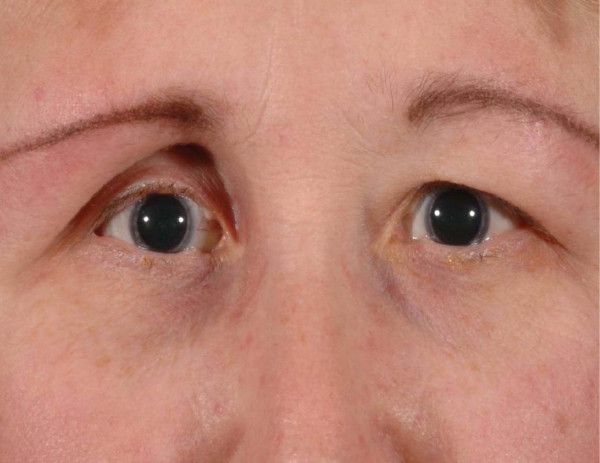
A colour photograph showing right enophthalmos.

**Figure 2:**
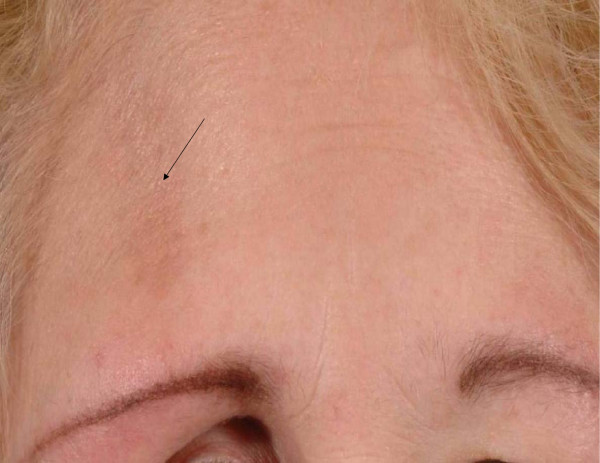
A colour photograph showing the "en coup de sabre" scar on the right forehead (marked by the arrow).

**Figure 3:**
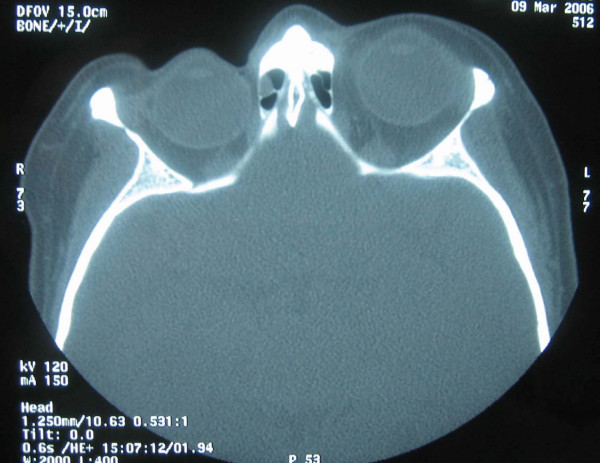
Axial CT scan demonstrating marked right enophthalmos.

A 64-year lady was referred to the oculoplastic clinic with painless and progressive shrinking of her right eye. She had no positive history for trauma or other medical problems. Her main concern was the disfiguring appearance of her right eye (figure 1). Her visual acuity was 6/6 in both eyes. There were no pupillary abnormalities or restriction of extra-ocular movements. Exophthalmometry measured 14 mm in right eye and 22 mm in the left eye. Both eyes measured an axial length of 22 mm in both eyes. There was no periocular paraesthesia. On closer examination she had a linear scar of 2 cm on her right forehead, which was missed during the preliminary examination (figure 2). A CT scan of the orbit showed no orbital fractures or any other intra orbital pathology (figure 3). The linear scar on her forehead, which was first discarded as an innocuous finding actually alludes to the early features in linear scleroderma, called **"en coup de sabre"**. She was referred to the dermatologist for further assessment.

## Discussion

The three basic structures that determine globe position are the bony orbits, the ligament system and the orbital fat. Any modification of the delicate balance between these three parameters will result in an alteration of the globe position. Enophthalmos is defined as the relative recession (backward +/- downward displacement) of the globe into the bony orbit [1]. The projection of the eye is most commonly measured relative to the orbital rim and/or in relation to the other eye. Enophthalmos greater than 2 mm relative to the other eye creates an observable cosmetic deformity [2]. Depending on the aetiology other significant morbidity may be associated [1].

**Scleroderma **may occur as a systemic disease or as a localised form [3]. Localised scleroderma presents in three clinical forms: generalised, morphoea (atrophic and sclerotic skin lesions), and linear scleroderma [3,4]. **Linear scleroderma **is characterized by localized fibrosis of skin, blood vessels, subcutaneous fat, muscle and sometimes bone. It primarily affects the population during the first and second decade [5]. Upper limbs are the most commonly affected but the fronto-parietal area of the forehead and scalp may also be involved initially. The skin is involved first and appears indurated. An ivory colored, band-like depression (en coup de sabre) of the frontoparietal region is characteristic.

Histopathogenesis shows two phases: an inflammatory phase and sclerotic phase [6]. Coarsened collagen bundles in the reticular dermis with perivascular lymphocytic infiltrates characterize the inflammatory phase. The skin appears indurated at this time. The collagen bundles become hyalinized, thus replacing subcutaneous fat and muscle, characterize the late sclerotic phase. Importantly, the elastic tissue is absent [6].

Ophthalmic manifestations may include atrophy, sclerosis, or inflammation of the eyelids, orbit, or globe. Patients can present with enophthalmos, lash loss, lid induration or tightening, periorbital edema, corneal opacities and thickening, keratoconjunctivitis sicca, fornix shortening, ocular myopathy or palsy, iritis, iris atrophy and heterochromia, retinal hemorrhages [3]. Other connective tissue disorders, lipoid dystrophies may accompany linear scleroderma. But these typically affect the fat and are bilaterally symmetrical.

## Conclusion

Linear scleroderma is an unusual cause of enophthalmos, however the presence of a linear scar on the forehead "en coup de sabre" should aid the examiner in making the accurate diagnosis.

## Competing interests

The author(s) declare that they have no competing interests. All authors declare no funding was required for the writing and submission of the manuscript.

## Authors' contributions

BSF and PSC prepared the first draft of the manuscript, participated in the analysis and interpretation of the data. KT and AEC designed the study. All authors contributed to the editing and revising of the manuscript and all authors have read and approved the final version.

## Consent

Full verbal and written informed consent has been obtained from the patient for the submission of this manuscript for publication and the accompanying images.
